# A Sharp Decline in Burden of Stroke in Rural China During COVID-19 Pandemic

**DOI:** 10.3389/fneur.2020.596871

**Published:** 2021-01-25

**Authors:** Jie Liu, Qiaoxia Yang, Xin Zhang, Qiuxing Lin, Yuan Yang, Dandan Guo, Wenjing Mao, Jun Tu, Zeping Liu, Jidong Li, Xianjia Ning, Jinghua Wang

**Affiliations:** ^1^Department of Neurology, Tianjin Medical University General Hospital, Tianjin, China; ^2^Laboratory of Epidemiology, Tianjin Neurological Institute, Tianjin, China; ^3^Key Laboratory of Post-neuroinjury Neuro-repair and Regeneration in Central Nervous System, Tianjin Neurological Institute, Ministry of Education and Tianjin City, Tianjin, China; ^4^Department of Cardiology, Tianjin Medical University General Hospital, Tianjin, China; ^5^Department of Neurology, North China University of Science and Technology Affiliated Hospital, Tangshan, China; ^6^Department of Internal Medicine, Tianjin Jizhou People's Hospital, Tianjin, China; ^7^Department of Neurosurgery, Tianjin Jizhou People's Hospital, Tianjin, China

**Keywords:** stroke, epidemiology, incidence, burden, COVID-19

## Abstract

This study aimed to explore trends in the burden from stroke associated with home quarantine during the COVID-19 pandemic. Patients with a first-ever stroke registered between January 1 and April 20 from 2010 to 2020 were included in this study. We compared the incidence and the rates of mortality, hospitalization, and diagnosis by neuroimaging for first-ever stroke among a low-income population in rural China during the study periods. Overall, 377 first-ever stroke patients were analyzed in this study period; men accounted for 59.2%. Compared with 2019, the incidence of first-ever stroke was 73.5% lower in 2020 (*P* < 0.001). The incidence of first-ever stroke was lower by 64.18% in 2020 than in the previous 5 years (*P* = 0.002) and by 65.42% in 2020 than in the previous 10 years (*P* = 0.001). Mortality from first-ever stroke in 2020 was not significantly different from that in 2019, but it was noticeably lower than that for the previous 5 and 10 years. However, rates of hospitalization and diagnosis by neuroimaging remained stable across the study period. These findings suggest that the home quarantine helped reduce outdoor activities at low temperatures, restrict gatherings, reduce alcoholism and high-fat diet, and lower pollution caused by factories. These changes were advantageous for helping high-risk groups to reduce the burden of stroke.

## Introduction

The World Health Organization officially classified the novel COVID-19 outbreak as a global pandemic on March 11, 2020. As of April 22, 2020, the cumulative number of affected patients worldwide was more than 2.3 million. Not only has COVID-19 had a serious impact on health and the economy worldwide, but it has also brought about major changes in people's lives. Severe acute respiratory syndrome coronavirus 2 (SARS-CoV-2) is extremely contagious. Over the last several months, governments across the world have issued self-isolation orders as a result of the COVID-19 outbreak. In China, the government issued a rule on January 23, 2020 to seal off the city of Wuhan, with a subsequent nationwide compulsory isolation order. These actions played an impressive role in blocking the spread of SARS-CoV-2.

Some studies have reported that patients with existing cardiovascular diseases may be at greater risk of developing severe COVID-19 (1, 2). An Italian study reported that cases of ischemic stroke decreased in the casualty department during the COVID-19 epidemic (3). However, the impact of isolation during the pandemic on chronic non-communicable diseases remains unknown, especially in low-income populations. Thus, we aimed to assess the effects of compulsory isolation during the COVID-19 pandemic on the burden of stroke in a low-income population in rural China.

## Methods

### Participants and Study Design

The study population came from the Tianjin Brain Study (4, 5), which began in 1985 as an on-going population-based study conducted in Tianjin, China, a township of Jizhou District that contains 18 administrative villages. The total population of this township was 14,285 in 2010 and 14,534 as of April 20, 2020; 95% are low-income farmers whose annual per capita income was <100 USD in 1991 and <1,000 USD in 2010.

Since January 23, 2020, strict measures for controlling the COVID-19 epidemic have been enforced and strengthened after the lock-down strategy implemented in Wuhan, China (6). On January 24, 2020, a first-level public health emergency response was carried out in Tianjin and the counties under its jurisdiction (7). Tourist attractions, entertainment venues, libraries, and schools were ordered to shut down. It was recommended that healthy residents stay at home and avoid mass gatherings to reduce the spread of the virus. People returning home from other provinces and cities were required to be in a centralized quarantine facility and to undergo medical observation for more than 14 days. Symptomatic patients and those suspected to be infected were required to receive treatment in an isolation ward (8, 9).

Tianjin is one of four municipalities in China, which includes 15 administrative districts. Overall, 136 patients were diagnosed with SARS-CoV-2 infections from 13 administrative districts in Tianjin during the study period. However, Jizhou District is one of two districts with zero infection cases of SARS-CoV-2 in Tianjin.

The research protocol was approved by Tianjin Medical University General Hospital Ethics Committee, and all patients provided written informed consent.

### Data Collection

Information on stroke patients was collected by the stroke surveillance network, which has been described previously (4). Briefly, in the Tianjin Brain Study, local licensed village physicians report stroke cases to physicians in community hospitals within 24 h of stroke onset. Physicians from the community hospital then visit the stroke patients' homes to confirm the stroke event and obtain information about the characteristics and clinical features. They then report confirmed and suspected stroke cases monthly to Tianjin Medical University General Hospital. Then, neurologists from Tianjin Medical University General Hospital use interviews to identify possible stroke events. The local licensed village physicians and the physicians from the community hospital are trained annually by a qualified neurologist.

Information was obtained by questionnaire about patients' age and education. Data regarding stroke subtypes, whether a diagnosis was made using computed tomography, and whether patients were hospitalized were collected within 24 h after patient admission was obtained by related medical records. In this study, patients were divided into three age groups: <45, 45–64, and ≥65 years. Education levels used years as the unit of measurement. Patients were divided into three education level groups: 0, 1–6, and ≥7 years.

### Definition of Stroke Events

First-ever stroke was defined as the first occurrence (i.e., without a history of stroke) of a rapidly developed focal (or global) disorder of cerebral function of vascular origin that lasted more than 24 h (10).

Stroke was categorized into three subtypes: hemorrhagic stroke, ischemic stroke (IS), and uncategorized stroke. Hemorrhagic stroke was defined as an intracerebral hemorrhage (ICH) or subarachnoid hemorrhage. IS was defined as a thrombotic brain infarction due to temporary or permanent occlusion of a feeding artery or of venous thrombosis. Uncategorized stroke was defined when patients' strokes could not be categorized as hemorrhagic stroke or IS or when there was a lack of evidence of neuroimaging. Patients with transient ischemic attacks, subarachnoid hemorrhage, and silent stroke, a kind of stroke detected only by imaging, were excluded in this study. Stroke was diagnosed by a professional neurologist based on typical clinical symptoms combined with imaging findings. Patients without neuroimaging in this study were diagnosed as having full clinical strokes with significant clinical symptoms and signs. For this study, all-cause mortality of stroke patients was used for mortality data.

### Statistical Analyses

Patients with a first-ever stroke who were registered between January 1 and April 20 for the years 2010 through 2020 were included in this study. Moreover, patients who died from stroke each year (i.e., 2010 through 2020) between January 1 and April 20 were also included in this study. Continuous variables, including age and education level, were analyzed with Student's *t*-tests and presented as means and standard deviations. Categorical variables are presented as frequencies and 95% confidence intervals (CIs); differences between groups were compared using chi-squared tests. All participants were categorized into three age groups (<45, 45–64, and ≥65 years) and three education groups based on the number of years of formal education (0, 1–6, and ≥7 years). The incidence of first-ever stroke events every year from 2010 to 2020 were calculated separately using the corresponding person-years. The age-standardized incidences were calculated with the direct method using the world standard population by 10 age groups: <35, 35–39, 40–44, 45–49, 50–54, 55–59, 60–64, 65–69, 70–74, and ≥75 years (11).

Changes in rates were calculated as follows: (rate in 2020—rate in reference years)/rate of reference years, and results are expressed as percentages. Differences in the incidence and rates of mortality, hospitalization, and diagnosis by neuroimaging between 2010 and 2020 were analyzed using the chi-squared test. Statistical significance was defined as *P* < 0.05. SPSS version 19.0 for Windows (IBM Corp., Armonk, NY) was used for analyses.

## Results

### Demographic and Clinical Characteristics of Patients With the First-Ever Stroke

Overall, there were 377 new cases between January 1 and April 20 for 2010 through 2020; men accounted for 59.2% (*n* = 223) of subjects. The average age was 65.7 years old overall (65.21 years for men and 66.42 years for women). The mean education level was 4.32 years (4.92 years for men and 3.44 years for women). There were 309 cases of IS (accounting for 82.0% of cases) and 62 cases of ICH (accounting for 16.4% of cases). Of these cases, 86.5% of patients were diagnosed by neuroimaging, but the rate of hospitalization was only 43% (Table 1).

**Table 1:** Characteristics of the first-ever stroke from January 1st to April 20th during 2010 to 2020 in Tianjin Brain Study.

**Category**	**Men**	**Women**	**Total**
Total	223 (59.2)	154 (40.8)	377 (100)
Age, means (SD), years	65.21 (10.86)	66.42 (12.81)	65.70 (11.70)
Education, means (SD), years	4.92 (3.27)	3.44 (3.26)	4.32 (3.34)
Age group, *n* (%)			
<45 years	10 (4.5)	6 (3.9)	16 (4.2)
45–64 years	97 (43.5)	65 (42.2)	162 (43.0)
≥65 years	116 (52.0)	83 (53.9)	199 (52.8)
Education group, *n* (%)			
0 years	40 (17.9)	60 (39.0)	100 (26.5)
1~6 years	123 (55.2)	71 (46.1)	194 (51.5)
>6 years	60 (26.9)	23 (14.9)	83 (22.0)
Stroke subtypes, *n* (%):			
IS	159 (71.3)	108 (70.8)	266 (71.1)
ICH	32 (14.3)	27 (17.5)	59 (15.6)
Uncategorized stroke	33 (14.3)	18 (11.7)	50 (13.3)
Hospitalized, *n* (%)			
Yes	90 (40.4)	72 (46.8)	162 (43.0)
No	133 (59.6)	82 (53.2)	215 (57.0)
Diagnosis by CT, *n* (%)			
Yes	190 (85.2)	136 (88.3)	326 (86.5)
No	33 (14.8)	18 (11.7)	51 (13.5)

*IS, ischemic stroke; ICH, intracerebral hemorrhage*.

### Incidence of and Mortality From First-Ever Stroke for 2010 Through 2020

**Figure 1:**
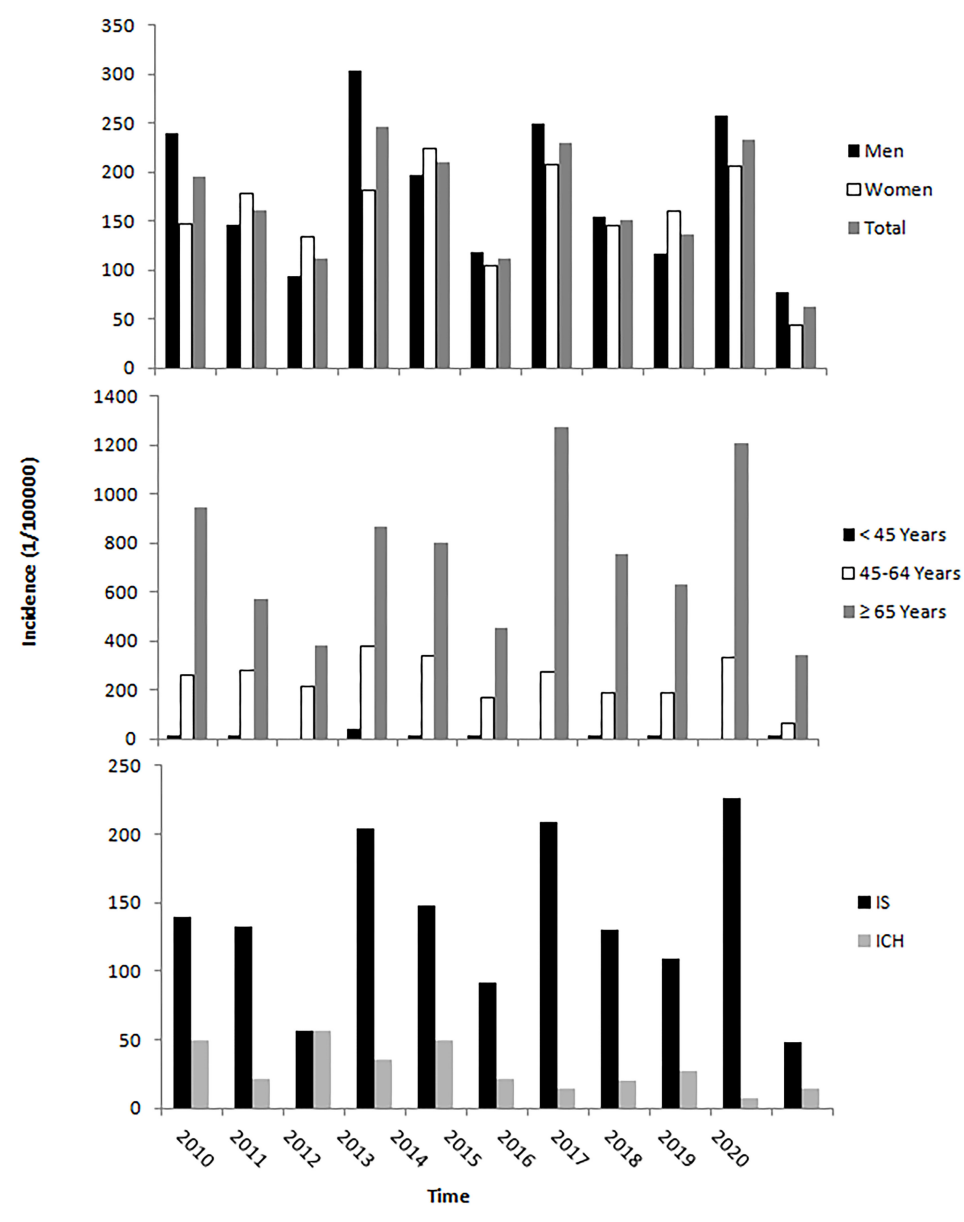
The incidence of the first-ever stroke from January 1 to April 20 during 2010 to 2020. The incidence of the first-ever stroke from 1st January to 20th April in 2020 was lower than that in the same time in previous years across sex, age, and stroke types.

Table 2 shows that the incidence of first-ever stroke in 2020 was 61.92/100,000 person-years overall (77.73 for men and 44.02 for women). The highest incidence was among those people aged 65 years and older (344.12/100,000 person-years). The incidence of IS was 43.68/100,000 person-years and of ICH was 5.66/100,000 person-years. Simultaneously, mortality per 100,000 person-years in this period was 6.88 overall (12.96 for men, 68.82 for people aged 65 years and older). There was a noticeably lower incidence in 2020 than in any other time from 2010 through 2019 (Figure 1). Similar trends were observed for mortality.

**Table 2:** The incidence and mortality of first-ever stroke from 1st January to 20th April during 2010 to 2020 (per 100 000 person-year).

**Category**	**2010**	**2011**	**2012**	**2013**	**2014**	**2015**	**2016**	**2017**	**2018**	**2019**	**2020**
**INCIDENCE**											
Total	196.01	161.19	112.65	246.51	210.51	112.26	229.84	151.01	137.15	233.64	61.92
**Gender:**											
Man	239.81	146.33	93.76	304.07	197.89	118.66	249.08	155.22	116.08	258.50	77.73
Woman	147.51	177.73	133.59	180.89	224.85	104.98	208.02	146.24	161.05	205.43	44.02
**Age group:**											
<45 years	12.38	12.41	0.00	38.28	12.70	12.55	0.00	12.23	12.22	0.00	12.06
45–64 years	259.35	280.66	214.50	379.51	336.28	168.95	273.22	187.11	186.95	333.89	62.63
≥65 years	948.77	569.62	379.51	865.80	801.48	451.32	1271.46	757.10	630.91	1207.24	344.12
**Stroke subtypes:**											
IS	75.67	87.90	27.30	131.54	94.36	57.93	117.65	94.58	74.88	149.92	43.68
ICH	36.35	8.50	35.74	26.74	31.72	17.36	9.37	13.38	17.46	4.11	5.66
**MORTALITY**											
Total	105.01	112.13	197.14	154.95	161.39	98.22	69.65	123.55	82.29	20.62	6.88
**Gender:**											
Man	133.23	159.64	214.30	185.09	184.70	92.29	117.99	168.15	64.49	25.85	12.96
Woman	73.76	59.24	178.12	120.59	134.91	104.98	14.86	73.12	102.49	14.67	0.00
**Age group:**											
<45 years	0.00	12.41	12.56	25.52	25.41	37.66	0.00	12.23	12.22	0.00	0.00
45–64 years	129.67	64.77	193.05	168.67	126.10	42.24	63.05	187.11	83.09	41.74	0.00
≥65 years	569.26	759.49	1138.52	742.12	924.78	580.27	445.01	504.73	441.64	67.07	68.82
**Stroke subtypes:**											
IS	33.58	49.66	75.13	61.43	43.79	60.55	9.37	51.61	41.85	13.73	0.00
ICH	8.51	10.42	40.58	25.80	35.81	17.36	9.37	21.10	14.47	0.00	5.66

*IS, ischemic stroke; ICH, intracerebral hemorrhage*.

### Hospitalization and Diagnosis by Neuroimaging for 2010 to 2020

Table 3 shows that the hospitalization rate for first-ever stroke in 2020 was 77.78%; overall, 83.33% were men and 66.67% were women. The hospitalization rate was 100% in patients aged <45 years old. Moreover, the rate of diagnosis by neuroimaging in 2020 was 100% across sex, age, and stroke subtype groups.

**Table 3:** Trends in the hospitalized rate and diagnosed by neuroimaging of first-ever stroke from 1st January to 20th April during 2010 to 2020 (%).

**Category**	**2010**	**2011**	**2012**	**2013**	**2014**	**2015**	**2016**	**2017**	**2018**	**2019**	**2020**
**HOSPITALIZED RATE**											
Total	46.43	43.48	25.00	45.71	50.00	68.75	78.79	45.45	25.00	52.94	77.78
**Gender:**											
Man	38.89	36.36	28.57	39.13	53.33	77.78	73.68	41.67	33.33	55.00	83.33
Woman	60.00	50.00	22.22	58.33	46.67	57.14	85.71	50.00	18.18	50.00	66.67
**Age group:**											
<45 years	100.00	–	–	33.33	100.00	100.00	–	100.00	100.00	–	100.00
45–64 years	66.67	53.85	40.00	50.00	43.75	75.00	92.31	55.56	22.22	50.00	66.67
≥65 years	26.67	33.33	0.00	42.86	53.85	57.14	70.00	33.33	20.00	55.56	80.00
**Stroke subtypes:**											
IS	50.00	44.44	33.33	44.44	47.62	66.67	88	36.84	33.33	58.06	75.00
ICH	71.43	100.00	25.00	80.00	83.33	100.00	100.00	100.00	–	–	100.00
**RATE OF DIAGNOSED BY NEUROIMAGING**											
Total	82.14	86.96	87.50	91.43	90.00	93.75	81.82	100.00	95.00	94.12	100.00
**Gender:**											
Man	83.33	81.82	85.71	91.30	93.33	100.00	68.42	100.00	88.89	100.00	100.00
Woman	80.00	91.67	88.89	91.67	86.67	85.71	100.00	100.00	100.00	85.71	100.00
**Age group:**											
<45 years	100.00	100.00	–	100.00	100.00	100.00	–	100.00	100.00	−	100.00
45–64 years	100.00	92.31	100.00	100.00	100.00	87.50	100.00	100.00	100.00	100.00	100.00
≥65 years	66.67	77.78	66.67	78.57	76.92	100.00	70.00	100.00	90.00	88.89	100.00

*IS, ischemic stroke; ICH, intracerebral hemorrhage*.

### Variation in Stroke Burden for 2020 vs. 2010–2019

Compared with the incidence of first-ever stroke in 2019, that in 2020 was 73.5% lower overall [*P* < 0.001; 69.93% for men [*P* = 0.006] and 78.57% for women [*P* = 0.008]]. There were similar trends in incidence for 2020 compared with that in the previous 5 years (2015–2019) and the previous 10 years (2010–2019), all *P* < 0.05. The overall incidence of first-ever stroke was significantly lower in 2020 than in the previous 5 years (64.18% lower; *P* = 0.002) and in the previous 10 years (65.42% lower; *P* = 0.001). These decreases were observed in both men (56.70 and 58.65% lower, respectively) and women (74.72 and 73.95% lower, respectively), but there was a greater difference observed for women. Moreover, the decreasing tendency was observed among those patients aged 45 years and over and with ischemic stroke across times. Of these, the greatest decrease was observed in patients aged 45–64 years, with a decrease of 81.24% from that in 2019 (*P* = 0.003). However, during the study period, the incidence of first-ever stroke remained stable for those patients aged <45 years and for those with ICH. There were no significant changes in mortality from first-ever stroke between 2019 and 2020, but mortality was noticeably lower than that for the previous 5 and 10 years across the sexes, age groups, and stroke types, except for patients aged <45 years and those with ICH.

The rates of hospitalization and diagnosis by neuroimaging remained stable across the study period (Table 4).

**Table 4:** Changes of the stroke burden from January 1st to April 20th in 2020 comparing to the same periods during 2010 to 2019 (%).

**Time** ** (Year)**	**Gender**			**Age group**			**Stroke subtypes**	
	**Total**	**Man**	**Woman**	** <45 years**	**45–64 years**	**≥65 years**	**IS**	**ICH**
**INCIDENCE**								
2019	−73.50^*^	−69.93^*^	−78.57^*^	–	−81.24^*^	−71.50^*^	−74.16^*^	0.12
2015–2019	−64.18^*^	−56.70^*^	−74.72^*^	63.30	−72.77^*^	−60.02^*^	−58.42^*^	−75.92
2010–2019	−65.42^*^	−58.65^*^	−73.95^*^	7.77	−76.09^*^	−56.25	−60.98^*^	−61.73
**MORTALITY**								
2019	−66.63	−49.86	−100.00	–	−100.00	2.61	−100.00	–
2015–2019	−91.27^*^	−86.16^*^	−100.00^*^	−100.00	−100.00^*^	−83.26^*^	−100.00^*^	−71.79
2010–2019	−93.87^*^	−90.34^*^	−100.00^*^	−100.00	−100.00^*^	−88.93^*^	−100.00^*^	−58.54
**HOSPITALIZED RATE**								
2019	46.92	51.51	33.34	–	33.34	43.99	29.18	–
2015–2019	137.89	142.74	123.45	99.00	110.12	156.65	27.51	62.49
2010–2019	56.17	70.23	31.04	50.00	21.57	83.70	42.50	57.70
**DIAGNOSED BY CT RATE**								
2019	5.88	–	14.29	–	–	11.11	–	–
2015–2019	8.00	10.14	5.36	–	1.82	13.43	–	–
2010–2019	10.12	11.19	8.77	–	1.61	19.35	–	–

*IS, ischemic stroke; ICH, intracerebral hemorrhage*.

* *P < 0.05*.

## Discussion

In this study, we assessed the effects of compulsory isolation during the COVID-19 pandemic on the burden of stroke in rural China in a low-income population. We compared the current incidence and rates of mortality, hospitalization, and diagnosis by neuroimaging for first-ever stroke during the COVID-19 pandemic in 2020 to those for previous years among a low-income population in rural China. We found that the incidence in 2020 was much lower than that in other time periods from 2010 through 2019 both in men and in women, in those aged 45 years and older, and for IS regardless of whether the reference was 2019, 2015–2019, and 2010–2019. Similar results were observed in mortality for 2015–2019 and 2010–2019. However, the rates of hospitalization and diagnosis by neuroimaging remained stable across the study period.

As the main cause of death worldwide, epidemiological trends of stroke have been receiving much attention. With the development of treatment and nursing technology, the fatality rate of stroke will gradually decrease and the patient's prognosis will be improved. In addition, the popularization of medical insurance will increase the detection rate of mild strokes. From 2010 to 2020, China's economy and medical level indeed have achieved rapid development. However, our previous research in this population found that first-ever stroke incidence increased by an average of 10.7% per 1,000 USD increase in overall per capita gross domestic product adjusted for purchasing power parity and by 12.0% per 1,000 Yuan increase in per capita net income (12). Moreover, the incidence of first-ever stroke in this study population showed an increasing trend from 1992 to 2018 (13–15). However, the burden of stroke from January to April 2020 was contrary to previous research trends, and the present study showed that the incidence and mortality of stroke decreased dramatically during the COVID-19 pandemic.

It is well-known that drinking alcohol and consuming a high-fat diet are risk factors for stroke (16–18). However, a previous study showed that drinking alcohol had positive effects on stroke onset (16). Another study reported that the cardiovascular benefits of low-moderate alcohol consumption perhaps might have been overestimated and that alcohol consumption may have no positive health effects (19). In addition, an animal experiment confirmed that prolonged consumption of a high-fat diet aggravates the condition of stroke (20). An Iranian case-control study demonstrated that the risk of stroke increased as high-fat diet consumption increased (21).

The Spring Festival is the biggest festival in China and occurs on the first day in the lunar calendar. The longest vacations in China are during Spring Festival, which often continues for 7 days. During that time, whole families not only hold a dinner party every day, but they also visit relatives, neighbors, and friends to pay a New Year call. This visitation and reunion results in drinking alcohol, consuming a high-fat diet, and extra excitement and tension. Consequently, Spring Festival is the peak time of stroke onset every year. Thus, home quarantine to a large extent caused people to avoid other people, drinking alcohol, and consuming a high-fat diet, which has been shown to be a risk factor for stroke (16–18).

Moreover, outdoor temperature seems to be related to cerebrovascular events risk. A previous study reported that the mortality of stroke was related with temperature, and cold temperatures were the main cause of increased stroke burden (22). Another national study in China also reported that both low and high temperature may increase the risk of stroke mortality, while the potential effect of cold temperature might last more than 2 weeks (23). Moreover, even a moderate decrease in temperature can increase the risk of ischemic stroke (24). The outdoor temperature in Tianjin from January to April is −3 to 4°C (25), which is the winter in Tianjin. Therefore, limiting outdoor activity and augmenting time spent indoors may contribute to the decreased incidence of first-ever stroke by inhibiting elevations in blood pressure. In addition, factories cut production or even shut down entirely during the COVID-19 pandemic, which indirectly reduced pollutant emissions. In the first half of 2020, the PM 2.5 concentration in Tianjin was 53 μg/m^3^, a year-on-year decrease of 7.0% (26). The risk of stroke has been shown to increase among people with long-term exposure to particulate matter PM 2.5 (27). Short-term exposure to particulates in air pollution has been shown to be associated with increased mortality from ischemic heart disease (2). Therefore, shutting down factories and production so that pollutant emissions are reduced may indirectly decrease stroke incidence and mortality.

Finally, a previous study has reported that viral infections can increase the risk of stroke and that the risk of stroke decreases by 24% after influenza vaccination (28, 29). A Spanish study demonstrated that hospitalization and mortality rates for stroke increased as influenza rates increased (30). Moreover, some studies have shown that the risks of morbidity and mortality were higher among older adults infected with viruses (31, 32), especially in January and February in North China. However, the spread of common influenza was effectively reduced due to compulsory isolation during the COVID-19 pandemic. In the present study, the incidence of first-ever stroke in 2020 was 81.24% lower in those aged 45–64 years and 71.5% lower in those aged 65 years and older compared with those for the preceding years.

During the period of the COVID-19 pandemic, the primary health care system in China played an important role in blocking transmission. Local rural physicians measured body temperature, handed out disinfection materials, and propagated the knowledge of prevention daily door-to-door. This practice was implemented to find new stroke patients early during this special period and thereby avoid delays in medical services due to isolation and allow patients to obtain timely medical treatment. In this study, it was observed that, compared with the same 4-months period in the past 10 years, the rates of hospitalization and diagnosis by neuroimaging in 2020 did not decrease during the COVID-19 pandemic. This result shows that home quarantine did not affect medical care-seeking behavior for stroke patients.

There are several limitations of this study. First, the study population was from 18 villages in Tianjin, which is not representative of the whole population in China. However, the large population study design and long study period may have reduced the impact of limited representation on the study results. Second, the observation period in 2020 only included the first 4 months. Thus, this result is not representative of the whole year. We would like to continue to follow to the end of the year and assess the whole impact of the pandemic on the burden of stroke in 2020. Finally, the data were analyzed using only descriptive statistics; we did not adjust for any potential confounders that could have affected the estimates of these changes. This statistical method could have partially affected results in this study.

## Conclusions

This paper is the first report to demonstrate trends in the incidence and mortality of stroke associated with home quarantine during the COVID-19 pandemic in a low-income population in China. The incidence of first-ever stroke in 2020 was much lower than that from 2010 to 2019. A similar trend was observed for mortality when compared with that in 2015–2019 and 2010–2019. Moreover, the rates of hospitalization and diagnosis by neuroimaging in 2020 remained stable across the study period. Thus, home quarantine may play a beneficial role in preventing stroke in rural China. In addition, even though COVID-19 was a huge challenge for the health system, medical services have not been affected in China. These findings suggest that the home quarantine helped reduce outdoor activities at low temperatures, the restrict gatherings, reduction alcoholism and high-fat diet, and decrease pollution caused by factories, etc. These changes may have been advantageous for helping high-risk groups to reduce the burden of stroke. It is crucial to explore simple and valid approaches to reduce the burden of chronic non-communicable diseases.

## Data Availability Statement

The raw data supporting the conclusions of this article will be made available by the authors, without undue reservation.

## Ethics Statement

The studies involving human participants were reviewed and approved by Tianjin Medical University General Hospital Ethics Committee. The patients/participants provided their written informed consent to participate in this study.

## Author Contributions

XN, JLi, and JW were involved in conception and design and data interpretation for this article. JLiu, QY, XZ, QL, YY, DG, WM, JT, and ZL were involved in data collection, case diagnosis, and confirmation for this article. JLiu, QY, and XZ were involved in manuscript drafting. JW was involved in data analysis for this article. JW and XN were involved in the critical review in for this article. All authors contributed to the article and approved the submitted version.

## Conflict of Interest

The authors declare that the research was conducted in the absence of any commercial or financial relationships that could be construed as a potential conflict of interest.
